# The Edinburgh Meeting

**Published:** 1898-07-30

**Authors:** 


### The Hospital

A Journal of

The Medical Sciences and Hospital Administration.

Vol. XXIV.—No. 618.] Edited by Sir Henry Burdett, K.C.B., and Solomon C. Smith, M.D., M.R.C.P. [July 30, 1898.

### The Edinburgh Meeting.

There can be no doubt that it must be difficult to follow Montreal, and that the pleasures of last year's visit to Canada must have somewhat spoilt many members of the British Medical Association for the less exciting delights of a meeting within the four seas. The travelling by sea and land, the new country, the fresh sights, the welcome which was extended to all, and the wonderful excursions which some enjoyed, made the Montreal meeting stand out far above its fellows, at least in the impressions which it left indented in the memories of those who went there. On the other hand, there is the fact that these were but a small proportion of the members—a small proportion even of those habitués who are to be seen again and again at almost every meeting of the Association, and that to those who did not go to Montreal the interval of two years has no doubt given an added piquancy to their reassembling, while, in any case, and to any people, no more attractive place of meeting within the confines of our island could be found than the city of Edinburgh.

As was said by Sir T. Grainger Stewart in his admirable address of welcome, not only have members of the Association been drawn to Edinburgh by a desire to increase knowledge, by the hope of meeting old friends and of becoming personally acquainted with those whose work has made their names familiar, but many have been drawn by other and very different attractions. Many have come to revisit the home of their student days and the haunts of happy youth. The old city is to them redolent of pleasant associations. I can fancy, he says, groups of fellow-students or brother-residents gathering to explore the wards or the class-rooms with which they were familiar, seeking out the site of the old infirmary, or the halls in which they had listened to Goodsir, Bennett, Simpson, or Syme. There are some who wish to see the house in which chloroform was first administered, or the wards in which the principles of antiseptic surgery were developed during most important years. But many come also attracted by the picturesque and the historical. "They wish to see the beautiful scenes which surround us, to explore the Castle, to trace the remaining fragments of the old city wall which was built as a defence against our ancient enemies of England, to visit Holyrood, to see the little back room in which the author of 'Waverley' achieved much of his finest work, or to make out the site of 'The Heart of Midlothian,' the home of Jeannie Deans, or of her faithful admirer the Laird of Dumbiedykes."

In speaking thus Sir T. Grainger Stewart adopted the proper tone in which to address such a meeting —a crowd of men of every shade of opinion, who, whatever else they might have come for, were all alike in this, that they were all out for a holiday. And as at Margate so at a meeting of the British Medical Association, holidays can be enjoyed in different ways. Some inquiring minds, mostly country practitioners, rush straight to the museum, from which they emerge with pockets full of pills and tabloids, and all sorts of odorous antiseptics, arms laden with advertising literature, and appetite destroyed by the doses of beef tea and other compounds they have been induced to swallow. Others stick steadily to "sections," at least for the first two days; others go seeing sights, or merely lounging about, enjoying the fact of being for once at nobody's beck and call; some hunt vigorously for invitations; a few apparently spend most of their time asking for letters at the post office, while a not inconsiderable number derive most of their enjoyment from their annual amusement of ballyragging the Council and abusing the Journal and all its ways. Year after year the old faces reappear, the old speeches are made, and the old indignation is poured out, and year after year the Council sits as tight as ever. For, after all, the objectors are but a little clique, and the mass of the members are indifferent. So that they receive their Journal, and have the satisfaction of feeling that they belong to a "great and powerful" organisation, and so that they now and then can join in its annual revels, they are mostly content to leave medical politics to those who, as they would say, have nothing better to do. Nevertheless, the ballyragging fraternity is, no doubt, a source of danger. Long, angry meetings get somewhat wearisome, the "indifferents," on whose support the Council more or less depends, go home to bed, and there always is a chance that a hostile vote may be passed. Fortunately, perhaps, for the Association, the Council's hide is thick. They take but little notice and all is well. So much for the holiday side. Turning to more professional topics, Sir Grainger Stewart dealt in turn with the relation of the profession to the Legislature in the prevention of disease; with the advancement of professional knowledge; with the training of our successors; with the necessity of guarding the portals of the profession; and with other matters, all of which he treated wisely and with a certain wit, but at too great a length for us to repeat with any fulness. But as to these things, are they not written in the Journal of the Association?

